# A quantitative assessment of the indirect impacts of human-elephant conflict

**DOI:** 10.1371/journal.pone.0253784

**Published:** 2021-07-12

**Authors:** Christie Sampson, S. L. Rodriguez, Peter Leimgruber, Qiongyu Huang, David Tonkyn

**Affiliations:** 1 Department of Forestry and Environmental Conservation, Clemson University, Clemson, South Carolina, United States of America; 2 Smithsonian Conservation Biology Institute, National Zoological Park, Front Royal, Virginia, United States of America; 3 Department of Biological Sciences Department, Clemson University, Clemson, South Carolina, United States of America; Wildlife Conservation Society, INDIA

## Abstract

Human-wildlife conflict has direct and indirect consequences for human communities. Understanding how both types of conflict affect communities is crucial to developing comprehensive and sustainable mitigation strategies. We conducted an interview survey of 381 participants in two rural areas in Myanmar where communities were exposed to human-elephant conflict (HEC). In addition to documenting and quantifying the types of direct and indirect impacts experienced by participants, we evaluated how HEC influences people’s attitudes towards elephant conservation. We found that 99% of participants suffered from some type of indirect impact from HEC, including fear for personal and family safety from elephants and fear that elephants will destroy their home. Despite experiencing moderate levels of indirect impacts from HEC at the community level, participants expressed attitudes consistent with supporting future elephant conservation programs.

## Introduction

People living in landscapes shared with elephants face both direct and indirect challenges resulting from human-elephant conflict [HEC; 1–3]. The direct costs of HEC for humans, which are more easily quantified and readily publicized, include crop, livestock and other property damages, injury, and loss of life. The indirect impacts of HEC are less tangible than physical damages. They include fear of attack, disruption of livelihoods and community activities, opportunity costs, resources lost to uncompensated activities such as guarding crops, pursuing compensation for crop and livestock loss, and harm to the psychological or social well-being of individuals or communities resulting from conflict-born injury [3–6]. Indirect costs of HEC are difficult to quantify, but are extremely important to assess to gain a full understanding of the impact of HEC on the lives of affected communities [7].

HEC severity varies with the location of the conflict and the status and culture of the individuals and communities involved, rendering HEC a complex and challenging issue with significant socio-economic, ecological, and political dimensions [8,9]. In addition, conflict events can vary in form and severity, from the disturbance of daily activities or minor crop raiding to injury or death of people and elephants [10], making sustainable “one-size-fits-all” solutions unlikely [11].

Wildlife management and conservation programs have begun to adopt more collaborative models that encourage engagement from all stakeholders [12,13]. Unfortunately, the wildlife agencies charged with executing conservation policy often lack the capacity to assess or mitigate HEC impacts on human welfare. Further, agencies that advocate for the needs of rural communities (e.g., social services, departments of human welfare) may be absent from the wildlife management decision-making process. These organizations also may not consider themselves stakeholders for HEC issues. In contrast, individuals from organizations focused on human- and wildlife-welfare both acknowledge that wildlife conservation projects must account for the needs of the affected communities as well [2,14].

HEC mitigation is further complicated because it frequently affects communities that are already disenfranchised. In many rural communities, direct and indirect HEC impacts are compounded by depressed economic situations, limited access to transportation and medical facilities, unmet basic needs and poor living conditions (e.g., malnutrition, access to water, sanitary housing). These poor conditions exacerbate the consequences of HEC on rural populations. Government corruption further complicate challenges to mitigating HEC because it can be more difficult to obtain government assistance [3,15].

As habitat for elephants continues to decrease, elephants and humans will be forced closer together and we can anticipate an increase in HEC [16]. This issue is often addressed through the combination of short-term mitigation strategies such as fencing and noise deterrents with long-term HEC reduction tactics such as education and land-planning [17]. However, understanding what factors contribute to local communities’ perceptions of HEC and the willingness of villagers to support elephant conservation may be key to finding solutions for sustainable coexistence and for the success of both short-term and long-term conflict mitigation strategies. Previous research indicates that a person’s gender [18–20], education level, perception of local government or NGOs in mitigation efforts [21], and even their aesthetic appreciation for species [18] can play roles in determining an individual’s willingness to support conservation. However, it is not clear how perception of conservation is affected by personal HEC experience. To understand conservation attitudes, we must also gain insight into community members’ experiences with elephants and the direct and the indirect impacts resulting from such experiences.

Previous studies have assessed the direct impacts of high levels of HEC on rural communities in Myanmar [22] and across Asia [*e*.*g*., 8,23]. A few studies have addressed indirect impacts qualitatively [3,4,24], but there is a lack of systematic, quantitative assessments of indirect HEC impacts and how they shape conservation attitudes. To address this gap, we studied indirect impacts of HEC facing villagers in Myanmar. Our research objectives were to: 1) determine and quantify which indirect impacts affect community members living near elephants; and 2) assess how HEC impacts the conservation attitudes of community members living alongside elephants.

## Methods and materials

### Questionnaire

We collected sociodemographic data on all individuals interviewed, including gender, age, ethnicity, education level, occupation, and length of residence in the village. We also assessed interviewee’s experience with and knowledge of elephants and HEC, and their beliefs about HEC and perceptions on best practices for deterring HEC. For people who had experienced direct impacts (*i*.*e*., property damage, personal injury, death of a family member) or were actively engaging in conflict mitigation (*i*.*e*., crop guarding), we asked about frequency and severity of associated indirect impacts. We also asked questions to better understand people’s perceptions relative to indirect impacts, general experiences with various indirect impacts, and attitudes towards elephant conservation.

The questionnaire was a mix of open ended, yes/no, and 5-point Likert scale questions. For the Likert questions, we employed visual aids to assist the respondents answering using the Likert scale [25]. We encouraged participants to complete the entire survey. However, some participants declined to respond to individual questions; the number of responses to each question is reported with the results. We pre-tested our questionnaire with Clemson University undergraduate students role-playing typical community member identities (*e*.*g*., farmer, grocer, daily laborer) and responding to the questionnaire. Once the questionnaire was finalized, it was translated into Burmese (Myanmar), and the translation was checked for accuracy by bilingual Myanmar team members who speak Burmese and English fluently. Our research teams were composed of Myanmar staff members from local environmental NGOs. Prior to implementation, the teams participated in a half-day training where they reviewed the questionnaire, practiced completing the form, and received instruction on avoiding biasing respondent answers by reading the questions exactly as written and separating participants as much as possible.

### Survey implementation

**Fig 1:**
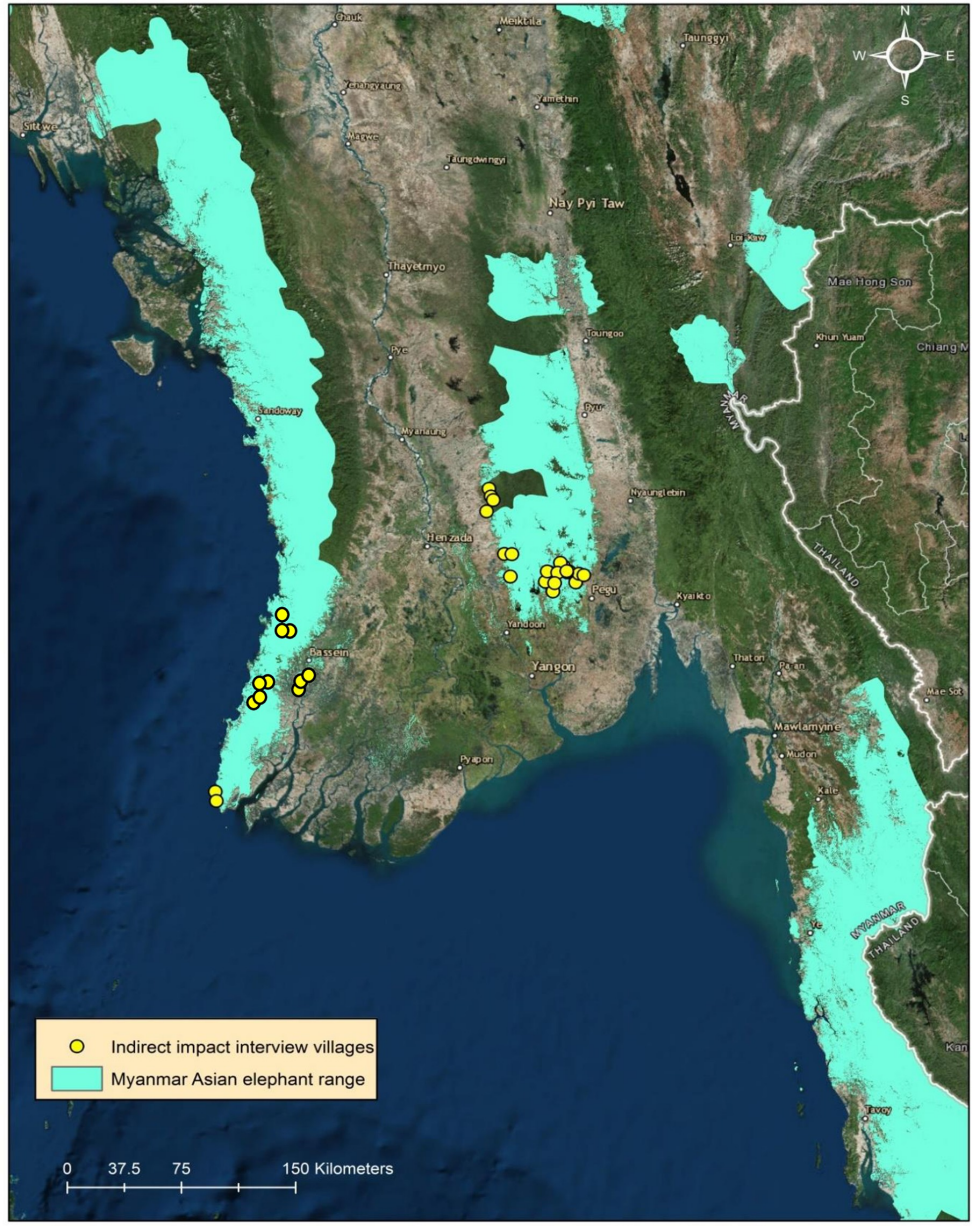
Locations of study sites in Myanmar. Dots indicate villages where interview surveys were conducted (May 2017-December 2018). Image source: World Imagery: Ersi, DigitalGlobe, Earthstar Geographics, CNES/Airbus DS. GeoEye, USDA FSA, USGS, Getmapping, Aerogrid, IGN, IGP, and the GIS User Community (accessed November 2017).

Our questionnaire was administered in Burmese in an in-person, oral interview format to adult male and female respondents from two rural areas of Myanmar: Bago-Yangon (24 villages), and Ayeyarwady (20 villages; Fig 1). These areas were chosen because of their location within the elephant range [26], and the presence of high HEC [27]. To maximize data collection, villages were selected based on their ease of access using available transportation, primarily bus or motorbike. Our data collection occurred between May—April 2017 and in December 2018.

At each village, the interviewer met with the village leader to ask permission to survey the community members. From that location, the interviewer approached the nearest residence to request an interview with an adult inhabitant. If no adults were present, or if the residents did not wish to participate, the interviewer moved to the next proximate house and every subsequent house until a willing participant was found. Once the interviewer had completed an interview, they skipped the next house and approached the second home for participation. This procedure was continued until a maximum of 40 people per village had been interviewed or until there were no more potential respondents present in the village. Both the questionnaire and the study design were approved independently by the Smithsonian and Clemson University Institutional Review Boards (HS16051 and IRB2014-187, respectively) prior to the start of the study. This work was conducted under a Memorandum of Understanding between the Smithsonian Institution and the Myanmar government (signed 2014).

### Data analysis

To quantify indirect impacts, we developed a scale designed to evaluate the severity of each participants’ experience, ranging from no indirect impacts to many indirect impacts suffered [28,29]. The questionnaire included six Likert statements intended to capture the different types and the severity of indirect impacts from HEC (*i*.*e*., fear for the safety of your family, loss of sleep, inability to travel). We summed the responses to the six 5-point Likert scale statements in Microsoft EXCEL [30], resulting in cumulative scores for each participant ranging from 6 (no indirect impacts) to 30 (all types of indirect impacts at a high severity). Participants who did not respond to all six Likert statements, or responded with “Don’t know” were excluded from the analysis. We were especially interested in examining the indirect impacts experienced by farmers because they can be particularly heavily impacted by HEC and farming is a common occupation. Therefore we posed two additional farming related Likert statements to farmers, and summarized the results of these eight statements, in a scale from 8 to 40, for the subset of farmers separately.

Similar to the indirect impacts scale, we developed a scale using ten Likert statements to capture participants’ support for elephant conservation in Myanmar, which resulted in a cumulative range from 10 (unlikely to support elephant conservation) to 50 (likely to support elephant conservation). For all negative Likert statements, we reverse coded the responses to align them in a common positive framing for the analysis. For example, responses to the statement “Conserving elephants is a waste of government effort”, were reversed so that the “disagree” responses were converted to “agree”, and the “agree” responses to “disagree”, effectively making the response as participant disagree with the statement ‘Conserving elephants is not a waste of government effort’.

To quantify participant’s experiences with direct impacts, we summed the number of types of direct impacts that participant had experienced at least once. The following eight direct impacts were included in the overall direct impact score: chased by elephant, home damaged, property damaged, crops raided, livestock killed by elephant, personally injured or family member or friend killed by elephant. Each direct impact was weighted equally regardless the number of times the participants had been impacted, so that each participant was assigned a score from zero to eight.

To better understand the relationship between direct and indirect impacts reported by participants and their elephant conservation attitudes, we conducted three linear regression analyses in R [31]. We examined if participants who experienced a greater number of direct impacts reported more severe indirect impacts or expressed more negative conservation attitudes towards elephants. We also determined if participants with more severe indirect impacts reported more negative conservation attitudes towards elephants.

## Results

We interviewed a total of 381 participants; 63 in the Bago-Yangon region and 318 in the Ayeyarwady with adults between the ages of 18 and 84; mean respondent age was 47. Most of the participants self-identified as male (70% male; 30% female). A majority of the participants identified with Bamar (64%), the dominant ethnic group in Myanmar, while the remainder of the respondents identified as Rakhine (24%), Kayin (10%), Karin-Bamar (1%), Bamar-Rakhine (<1%), Chin (<1%), or Rakhine-Kayin (<1%), and two respondents refrained from answering. On average, people indicated having lived in their current village for 31 years. Most participants had attended school (85%), generally, between the ages of 5 and 12, receiving more than the United Nation estimate of 5 mean years of schooling across the country [32]. The most common occupation was farming (73%), and these farmers (n = 217) reported an average annual income of 1,720,737 kyat ($1,433 USD).

### Experiences with HEC

Local community members in areas with HEC are frequently exposed to elephants. The majority of participants (90%) had seen an elephant in the wild at least once, and 34% of the individuals reported being chased by a wild elephant. A common indirect effect was the need to share resources with someone who had experienced a direct HEC event to help with recovery. This occurred either to help a family member (10%; primarily food) or to help a non-family community member (14%, primarily food and labor).

### Elephant knowledge

Participants rated their knowledge about elephants as very low: only 12% rated themselves as very knowledgeable about elephant behavior and only 2.6% claimed to be very knowledgeable about elephant biology. Subsequent responses about elephant natural history, behavior and conflict (Table 1) indicate that respondents possess at least some knowledge of elephants, with 75% of the respondents correctly answering six or more of the 11 questions. It should be noted that 82% of the participants correctly indicated that elephants had lost habitat because of human activities, while only 42% knew that Asian elephants are endangered.

**Table 1:** Local people’s knowledge about Asian elephant natural history, behavior, and conservation in the Bago-Yangon and Ayeyarwady regions of Myanmar from May 2017 to December 2018.

Elephant knowledge questions (Correct answer)	N	% that responded correctly	Number of "I don’t know" responses
Elephants kill other animals to eat the meat (N)	378	93%	38
Elephants eat tree bark, roots, and leaves (Y)	379	97%	3
During harvest season, elephants eat only human crops (N)	372	45%	17
Only male elephants kill people (N)	378	57%	94
Only adult elephants raid crops (N)	377	43%	22
Elephants have lost habitat because of human activities (Y)	376	82%	2
Female elephants live in herds (Y)	350	48%	84
Elephants have emotions like love, anger, and grief (Y)	375	83%	38
Elephants have a good memory and can remember other elephants (Y)	379	93%	22
Asian elephants are endangered (Y)	344	42%	86
Asian elephants are legally protected in all countries (Y)	335	73%	35

Response options included yes (Y), no (N), or “I don’t know”, with percent of participants that responded correctly indicated in the table. Participants who responded “I don’t know” were excluded from the analysis.

### General beliefs and best practices of HEC

Only a small fraction of the participants, 16%, indicated that they know how to act when near elephants to avoid conflict (Table 2). Ninety-two percent of participants believed that elephants will eat crops whenever they are available, and 37% believed elephants would always attack humans when possible. Very few individuals (3%) responded that they were angry with the Myanmar government for not providing compensation for property damage caused by elephants, or when someone is injured or killed due to HEC.

**Table 2:** Participant’s beliefs about human elephant conflict in the Bago-Yangon and Ayeyarwady regions of Myanmar from May 2017 to December 2018.

Belief Statement	n	Average	SE
All wild elephants are dangerous	334	3.93	0.05
Adult male elephants cause the most conflict (including both crop raiding and injury to humans)	272	3.81	0.06
When possible, elephants will always attack people	313	2.35	0.06
When possible, elephants will always eat crops	322	3.63	0.19
HEC has gotten worse in my area since permanent water sources in my area were constructed	269	3.15	0.06
As agriculture expands and human populations grow in my area, HEC will get worse here	305	3.81	0.05
I know how to act around elephants to avoid potential conflict	252	3.11	0.06
It is best to scare elephants away using lights and firecrackers	309	3.16	0.07
Any elephant seen near a village must be scared away even if they are not causing damage/harm	302	3.13	0.07
I am angry that the government will not compensate me for elephant damage to my property	326	2.46	0.06
I am angry that the government will not compensate me or my family if one of us is injured by an elephant	326	2.55	0.06
I am angry that the government will not compensate me or my family if one of us is killed by an elephant	323	2.56	0.06

Based on average of the responses by 381 interviewees to 5-point Likert scale questions, ranging from 1 = Strongly disagree to 5 = Strongly agree and the standard error (SE).

### Damage to home or property

Reports that elephants had damaged their home were relatively common among participants (14%), while 7% reported that elephants had destroyed a tree or crop guarding hut. Of the people whose homes had been damaged, most had been able to repair the damage within one week to two months. However, two respondents indicated that it had taken a year to rebuild. The average cost to rebuild a damaged home was 587,647 kyat (approx. $490 USD, n = 17). Five participants from four different villages reported that their livestock (i.e., cattle, buffalo, ox) had been killed by elephants, and 63 participants from 12 different villages (including the four villages listed above) reported that at least one of their neighbors had lost livestock due to HEC.

### Crop guarding and farming in an elephant landscape

Growing crops in areas with elephants pose significant challenges to farmers. Crops grown by participants in the study area are primarily rice paddy (60% of farmers) which is harvested once per year, followed by cashew (24%), and betel leaf (11%). Of the 278 participants that were self-identified farmers, 49% reported that they guard their crops to prevent crop raiding by elephants. Half of the crop guarding farmers (50%) indicated that they stay awake all night, often spending the night in their field (55%) to protect their crops against elephants. A majority (64%) also received help crop guarding either from their spouse, another family member, and/or a neighbor. Methods of deterring elephants from crop raiding included making noise (e.g., shouting, playing guitar), throwing firecrackers, and shining flashlights. More than half of the farmers that crop guarded (53%) reported that crop guarding takes time away from caring for their children. Sixty percent reported that guarding crops prevented the guard from getting enough sleep, and 61% indicated that guarding crops made the guard more susceptible to illness. A quarter of all farmers (25%) reported that they worked with their neighbors more on preventing crop loss than any other activity, and 20% believed that it improved social bonds within their village. Only 28% of farmers believe that crop guarding was effective at reducing actual crop loss from elephants. A quarter of the farmers (25%) reported that they had not expanded the farm because they were worried elephants will destroy it, and 20% indicated that they would grow alternate crops such as coconut, mango, cashew, or banana if elephants were not present.

### Injury by elephant

Eight participants reported being injured by an elephant, however only three of those were comfortable discussing their experience. All three reported that they were more afraid of elephants for themselves and for their families since the injury occurred, and that they worried for their ability to care for their children due to this injury. Two of the three reported that they felt more susceptible to illnesses due to the injury, while one reported continuing to lose sleep due to ongoing pain and post-traumatic stress. Furthermore, two of the three participants reported incurring significant medical expenses, 100,000 kyat ($83 USD) and 200,000 kyat ($166 USD) respectively, while at the same time being unable to work. All three injured participants reported that their spouses had to work extra and take on more of the childcare responsibilities, which they believed led to a decrease in quality of life for their spouses. Similarly, two of the three reported that a friend or family member cared for them while they were injured, their caretaker lost income, sleep and time with their own children while tending to them.

### Family member killed by an elephant

Eleven participants (3%) had experienced deaths in their immediate family due to HEC, including the loss of siblings, a parent, a spouse, children, nieces, and a grandchild. Five people reported that they had to work more to make up for the income loss resulting from the death of a wage-earning family member. Participants also reported that this loss harmed their health, caused a loss of sleep, and made them more susceptible to illness. This additional work also reduced the amount two participants were able to spend caring for their children. Eight people reported they worried more for their family’s safety after losing a member to HEC. Nine people worried more about their ability to care for their children and eight people worried about their ability to provide food and shelter for their family since their loss. In addition, nine people reported that they feared elephants more and eight indicated they lost sleep due to this increased fear. When asked if they had any additional comments in relation to the loss of their family member, one participant reflected that their “family is suffering because of elephant”, while another commented “nothing special because it is luck”.

### Caretakers of people injured by an elephant

Nine people reported acting as a caretaker for someone injured by an elephant, for neighbors (n = 6), immediate family members (n = 2) or unspecified relation (n = 1). Periods of caregiving ranged from one day to one year, although one participant indicated the need to provide lifelong care to his wife (5 years at the time of the study) due to her elephant-related injuries. All caretakers indicated that the person injured was more afraid of elephants after the incident, while most indicated that the injured person lost income and sleep due to the incident (n = 8), and worried more about their ability to provide resources such as food and shelter for their families (n = 8). They also reported the injured person felt they were more susceptible to illnesses (n = 5) and were unable to care for their children while inured (n = 5). Three people caring for their neighbors took on additional work to help pay for medical expenses, and the spouses of those caretakers worked more to cover the loss of income while their husbands or wives cared for their injured neighbor. Five of the nine participants reported that acting as a caretaker made them more worried for the safety of their families from elephant encounters, and for their ability to provide for their family in the future due to HEC. Six participants reported they personally feared elephants more as a result of caretaking, with three of these six reporting a loss of sleep due to this fear. Furthermore, four of the nine participants reported losing sleep due to the additional demands of being a caretaker.

### Impacts of HEC on children

Two hundred and sixty-eight of the participants reported they had children or grandchildren 18 years old or younger living in their home, but many participants were not comfortable discussing these children in the context of HEC with the research team. Sixty-three percent of participants who did respond (n = 74) indicated that their children were constantly afraid of encountering elephants outside of their village, and 55% believed that their children have a lower quality of life because they live near elephants. Similarly, 54% of participants (n = 74) said their children had a lower quality of life because they were afraid of elephants. Twenty-six percent (n = 42) reported that their children were unable to attend school when elephants were near their homes. One person reported that while he was rebuilding his home, his son was unable to attend school and that his son’s performance in school suffered. Another reported that although his child was still able to attend school during the rebuilding process, their performance similarly suffered.

### Fear vs. expectation of HEC

Participants rated their fear of a HEC event occurring higher than the expectation such an HEC event would actually occur in every category (Table 3). Participants also believed it was more likely that they themselves would actually be injured than their spouses or their children.

**Table 3:** Fear versus expectation expressed by residents in Bago-Yangon and Ayeyarwady regions of Myanmar from May 2017 to December 2018.

Fear and expectation statements	N	Average	SE
I am afraid elephants will physically injure me	341	4.38	0.04
At some point in the future, an elephant will physically injure me	315	4.08	0.06
I am afraid elephants will physically injure my spouse	327	4.28	0.04
At some point in the future, an elephant will physically injure my spouse	316	3.94	0.06
I am afraid elephants will physically injure my child/ren	328	4.30	0.04
At some point in the future, an elephant will physically injure my child/ren	312	3.93	0.06
I am afraid elephants will physically injure my neighbor/another person I know	326	4.26	0.03
At some point in the future, an elephant will physically injure my neighbor/another person I know	307	3.92	0.05
I am afraid an elephant will destroy my house	331	4.13	0.05
At some point in the future, elephants will destroy my house	317	3.81	0.06
I am afraid an elephant will destroy my crops	324	4.04	0.06
At some point in the future, elephants will destroy my crops	313	3.81	0.07
I am afraid an elephant will kill my livestock	329	3.56	0.07
At some point in the future, elephants will kill my livestock	318	3.38	0.07

Based on average of the responses by 381 participants to a 5-point Likert scale, ranging from 1 = Strongly Disagree to 5 = Strongly Agree and the standard error (SE).

### Indirect impact score

Indirect impacts affected 99% of participants in our study. The most common indirect impacts experienced by the participants were fear for their own or their families’ safety (85%, Table 4), and fear that an elephant would destroy their house (68%). A total of 268 participants responded to all six Likert statements, and were therefore included in the analysis. Participant’s scores for the indirect impacts scale ranged from 26 to 50, with an average was 23.4 (on a scale of 6 to 30), indicating they were experiencing a moderate to high amount and severity of indirect impacts from HEC. Similarly, the participants who self-identified as farmers and responded to the six Likert statements asked of all participants as well as the additional two farming specific statements, had an average indirect impacts score of 32.4 (on a scale of 8 to 40; Table 5).

**Table 4:** Indirect impacts experienced by participants (n = 381) in the Bago-Yangon and Ayeyarwady regions of Myanmar (May 2017-December 2018).

Indirect impact statements	N	Average	SE
I am afraid elephants will physically injure me	341	4.38	0.04
I am afraid an elephant will destroy my house	331	4.13	0.05
I fear that I will not be able to support my family if my property sustains damage from elephants	329	3.60	0.07
I am afraid elephants will physically injure my neighbor/another person I know	326	4.26	0.03
Sometimes I am afraid to travel outside my village because of elephant activity in the area	315	3.72	0.05
I lose sleep because I am worried that conflict with elephants will negatively impact my ability to perform well at my primary occupation	311	3.17	0.08

These statements were used to create a study-wide scale to evaluate the extent and severity of indirect impacts represented by an average of the responses to the 5pt Likert scale which ranged from 1 = Strongly Disagree to 5 = Strongly Agree and the standard error (SE).

**Table 5:** Indirect impacts experienced by farmers (n = 278) and used to create a farmer specific indirect impact scale in the Bago-Yangon and Ayeyarwady regions of Myanmar (May 2017-December 2018).

Indirect impact statement (Farmers)	N	Average	SE
I am afraid elephants will physically injure me	251	4.79	0.38
I am afraid an elephant will destroy my house	245	4.52	0.39
I am afraid elephants will physically injure my neighbor/another person I know	241	4.73	0.39
I fear that I will not be able to support my family if my property sustains damage from elephants	230	4.33	0.42
Sometimes I am afraid to travel outside my village because of elephant activity in the area	227	3.81	0.06
I lose sleep because I am worried that conflict with elephants will negatively impact my ability to perform well at my primary occupation	216	4.00	0.45
I am afraid an elephant will destroy my crops	244	4.68	0.39
I am afraid my family and I will not have food because elephants will destroy my crops	238	4.47	0.40

Average of the responses to the 5 pt Likert scale which ranged from 1 = Strongly Disagree to 5 = Strongly Agree and the standard error (SE).

### Elephant conservation attitudes

Local people in HEC landscapes continue to show overall positive conservation attitudes to elephants in Myanmar. A total of 232 of the participants responded to all ten Likert statements included in the conservation attitude assessment. Their responses reflected positive attitudes towards elephant conservation (Table 6), as indicated by an average attitude score of 38.6 on a scale from 10 to 50.

**Table 6:** Elephant conservation attitudes of 381 participants in the Bago-Yangon and Ayeyarwady regions of Myanmar (May 2017-December 2018).

Elephant attitude statements	N	Average	SE
Elephants should be protected by law in Myanmar	354	4.21	0.03
It is important to protect elephant habitat in Myanmar	351	4.23	0.03
Elephants are important to the ecosystem	296	3.68	0.05
Elephants are important for religious reasons	337	4.02	0.03
Elephants are an important part of Myanmar’s culture	336	4.02	0.03
It is a waste of money for the Myanmar government to protect elephants*	311	2.23	0.04
People who poach elephants should be punished	359	4.12	0.06
My family and I benefit from conserving elephants	347	2.95	0.06
All the people in Myanmar benefit from conserving elephants	350	3.37	0.05
We should remove all elephants from Myanmar*	322	1.97	0.06

These 5pt Likert statements were used to create a scale to evaluate the overall elephant conservation attitude and ranged from 1 = Strongly Disagree to 5 = Strongly Agree. Negatively framed statement, indicated with a “*” were reverse coded before inclusion in the conservation attitude score.

### Direct impact scores

Individual participants reported experiencing from zero to five direct impacts, with an average of two each. The most common direct impacts reported the participants included that their crops had been raided by elephants (n = 198, S1 Appendix), that a someone they knew had been injured or killed by an elephant (n = 156), or that they themselves had been chased by an elephant (n = 128).

### Relationship between conflict and conservation

Indirect impacts significantly increased with severity of direct impacts based on the results from the linear regression (Table 7). We found that as the number of direct impacts participants experienced increased, there was a small but significant increase in the severity of indirect impacts they reported. We found no significant relationships between direct impact and conservation attitude or indirect impact and conservation attitude.

**Table 7:** Effects of direct and indirect impacts on conservation attitude in the Bago-Yangon and Ayeyarwady regions of Myanmar (May 2017-December 2018) based on the linear regressions from responses of the 203 participants who answered all applicable Likert statements.

Model	Predictor variable	Estimate	Std. Error	P-value	R-squared
Indirect Impact Score ~ Direct Impact Score	(Intercept)	20.816	0.428	<2e-16	0.130
	Direct Impact Score	1.115	0.204	1.27E-07	
Conservation Attitude ~ Direct Impact Score	(Intercept)	37.814	0.483	<2E-16	0.021
	Direct Impact Score	0.480	0.230	0.038	
Conservation Attitude ~ Indirect Impact Score	(Intercept)	35.013	1.710	<2E-16	0.022
	Indirect Impact Score	0.159	0.074	0.033	

## Discussion

Indirect effects of HEC are significant and their impact on the well-being of rural populations is likely to be highly significant. These communities are frequently disenfranchised and HEC experiences may future disadvantage them as well as lead them to resent other parts of society and government. We provide an overview of the significant side effects we believe are linked to the indirect impacts described in our study and provide suggestions for how to address these issues in the future to reduce HEC impacts, conserve elephants, and assist rural communities in elephant range counties. Our finding that everyone we interviewed, with the exception of one participant, reported experiencing indirect impacts indicates the need for more research in this area, and a potential shift in mitigation strategies towards addressing these less tangible but more widespread challenges that come with living alongside elephants.

### Relationship between experiences with HEC and conservation

We were surprised to find participants reported generally positive elephant conservation attitudes regardless of the amount of conflict experienced. This is potentially due to the importance of elephants for cultural and religious reasons [22], or because some amount of HEC is expected and accepted by local communities. Previous research also suggests that communities in Myanmar believe that other factors, such as poverty, have a greater effect on their well-being than HEC [22]. The weak relationship between the number of direct impacts participants reported and their indirect impact score could be attributed to the lack of variability in the number of direct impacts participants reported experiencing, where most of the participants cited two or fewer direct HEC events. Conducting non-random sampling to seek out participants that have experienced greater numbers of impacts in the future may help to clarify any correlations not captured in our sample.

### Damage to home or property

Although most respondents were able to repair their homes quickly, the results of damage to a home can extend beyond the physical destruction to impair a person’s sense of security and safety [33]. The degree to which the victim experiences loss of sentimental possessions, increased demand for additional time and money to repair the home, and strains on the ability to work afterward can further increase the psychological distress [34]. While little research has been done on the specific psychological effects of losing a home during a HEC event, victims of home damage or loss during a natural disaster or residential fire experience a broad range of psychosocial problems including anxiety disorders, depression, post-traumatic stress disorder, and an increase in the occurrences of domestic violence and divorce [35,36]. Further, the unexpected destruction of a family shelter can be traumatic for both adults [35] and children [37], with symptoms of distress lasting for up to six months, even when no physical injuries resulted from the event [38,39].

### Crop-guarding and farming in an elephant landscape

Effective crop guarding relies on a person’s ability to remain vigilant, to detect and respond to the unpredictable event of an elephant approaching the crop, and primarily occurs at night. Farmer participants reported that crop guarding prevented them from obtaining enough sleep, which is congruent with previous research on impacts of HEC on farmers in India [3,5]. Vigilance is the function most highly impaired by insufficient sleep, compared to other complex cognitive functions [40,41]. Impaired vigilance is increased when a person extends their waking hours into nighttime against the human body’s normal circadian rhythm [42,43], and has been demonstrated to be a major factor contributing to the increase of accidents that occur at night [41]. In Nepal, researchers found that 72% of the HEC events that resulted in human fatalities occurred during the late evening and night [44]. One possible factor contributing to this could be the inability of the farmers to maintain vigilance while guarding their fields during times of peak elephant activity.

Not only can crop-guarding at night lead to chronic sleep deprivation, threatening the farmers’ mental and physical wellness [45,46], but it can also expose them to additional health threats [47]. Myanmar, and much of Southeast Asia, is host to a variety of mosquito-borne diseases including dengue fever, malaria, and zika. Exposure risks are greatest in the evening and at night when the highest densities of mosquitoes are present, and inopportunely when farmers are reportedly guarding their crops. In addition, a study in India concluded that water contained in elephant footprints was found to be the second most prominent breeding ground for the malaria carrying mosquito *Anopheles baimaii* [47].

One potentially positive consequence of HEC is that 20% of the farmers surveyed reported that working with their neighbors to mitigate crop raiding also improved social bonds in their community. The common enemy effect, here crop-raiding elephants, encourages cooperation amongst unrelated individuals in pursuit of a common goal [48]. Camaraderie in the face of adverse situations such as war has been shown to be particularly strong when the people involved share similar qualities such as socioeconomic status, religion, or ethnicity [49]. And while such camaraderie can have short-term positive effects, such as increased likelihood of sharing food between individuals or emotional support in the face hardships [49], it can also have long-term positive effects, such as reducing the severity or onset of PTSD in the event of an extremely traumatic event such as injury or death of a loved one [50].

### Human injury, death, and caretaking

Previous studies suggest that animal attacks can leave the human victim not only physically disabled but also suffering from debilitating fear and mental stress [3–5]. Post-traumatic stress disorder [PTSD; 51] is commonly suffered by victims of life-threatening events and can include symptoms such as depression, hostility, hypervigilance and insomnia. Both the physical and mental consequences of injuries resulting from HEC can hinder or prevent the victim from future employment, thus increasing the financial strain on the victim and their family [3,4].

Caretakers of victims who have experienced a traumatic event or been severely injured can also endure PTSD themselves [51], be subject to the harmful and aggressive behaviors that victims exhibit post-HEC incident [5], and suffer from compassion fatigue [52,53]. Caretakers who exhibit compassion fatigue can suffer symptoms including negative cognitive, emotional, and physical reactions that lead to a decreased level of concern and empathy for the victim of the attack, negative feelings towards the victim, and physical and emotional exhaustion [54]. A majority of the caretakers interviewed in this study reported experiencing increased stress, loss of sleep, and higher workloads, which can make them more susceptible to PTSD or compassion fatigue.

Similar to studies that used focal groups to assess the impacts of losing a family member to HEC [4,5], the participants in our study indicated that they experienced increased levels of fear towards elephants, difficulty sleeping, and an increase in worry felt for their remaining family’s safety after the conflict. These previous studies, whose methodology allowed for a deeper exploration of the consequences of HEC, also revealed that surviving family members of victims killed by elephants in India and Bangladesh experienced emotional trauma resulted in depression, anger, humiliation, feelings of helplessness, and of being overwhelmed with the additional responsibilities and financial burdens [4,5]. Poverty and poor mental health care support can exacerbate the problems faced by people who lose a loved one to HEC [5]. Five participants reported that after a family member’s death, they had to take on additional work to support their family. When the primary earner in the family is injured, usually the male head of household, the burden of supporting the family typically shifts to the female head of household, and potentially the victim’s children [3–5].

### Impacts of HEC on children

While chronic stress and fear can be damaging to adults, they can also be harmful to children growing up in areas where they feel threatened by HEC. Research has shown that chronic stress can change the structures and functioning of prefrontal cortex [55] and have lasting effects throughout a person’s lifetime [56]. Children who live in chronic fear often lose the ability to differentiate between safe and threatening situations, which inhibits their ability to learn and promotes the development of anxiety disorders [56,57]. Limited access to mental health care, either because it is not available locally or it is too expensive, can further marginalize children living in proximity to conflict species.

### Fear vs. expectation of HEC

Fear is an important part of biological preparedness in the event of danger [58,59 but see 60] and in reacting appropriately to animals that may cause harm [61]. However, fear of elephants may lead to the overestimation of the likelihood of actually encountering elephants and experiencing HEC [62]. In our study, nearly 60% of participants believed their spouse would be injured by an elephant, yet < 1% of participants (n = 2) reported their spouse actually was injured or killed during an HEC event. Likewise, 87% indicated they believed their children would be victims of HEC, but <1% (n = 3) had a child injured or killed as a result of HEC. Working with rural communities to manage the perception of danger while maintaining appropriate safety precautions will be an important aspect of outreach as conservation efforts continue.

### Elephant conservation

Our results revealed differences in stakeholder priorities regarding HEC mitigation focuses and potential avenues for the Myanmar government and other agencies to explore when developing elephant management and conservation policy initiatives. For example, aside from issues of safety, when evaluated as a whole, participants were most worried about elephants damaging their homes, whereas farmers expressed fears that elephants would destroy their crops. Despite all participants experiencing some form of indirect impacts from HEC, they expressed strong support for legal protection of elephants and their habitat, and were generally very supportive of elephant conservation. While our results showed variability in the amount of HEC experienced, future studies that focus on sampling participants who have experienced extremely high levels of HEC (e.g., indirect score > 40, direct score ≥ 4) may reveal stronger relationships between the levels of indirect and direct HEC experienced and conservation attitudes.

The small number of participants who indicated they knew how to act around elephants suggests there is ample opportunity for educational outreach by NGOs and other agencies to instruct community members on tactics for avoiding HEC. Communities in the study areas generally use fire crackers or shout at elephants to scare elephants away even if they are not causing damage, which can increase confrontations and aggressive behavior between humans and elephants [63]. Helping the community to understand why it is important to conserve elephants and what terms such as ‘endangered’ mean in a global context may build support for management programs aimed at sustaining elephant populations over the long-term. As long as humans and elephants coexist in a landscape there will be conflicts [63], therefore working with the communities to help them understand elephant behavior and ecology can increase tolerance for benign interactions, improving coexistence between the two species. Further, incorporating both human and elephant behavior into mitigation strategies can help managers and other stakeholders develop more effective conservation policies [64,65].

## Conclusions

One fundamental challenge in conservation is that increasing the population size of a target species often increases the negative consequences for humans who share the landscape. Local support of species conservation programs is critical to their long-term success. Previous research has demonstrated that communities will not support long-term conservation goals if those goals infringe on the community’s ability to procure resources needed for their survival in the short-term [66]. Although our results show that communities within the study area are inclined to endorse elephant conservation efforts, which would seek to maintain or increase current population sizes, our results highlight areas researchers can work to address indirect impacts before an increase in the elephant population were to take place.

Our quantitative assessment provides scale and critical contextual information about the rate at which different indirect impacts are being experienced, and thus complements previous qualitative research on indirect impacts [*e*.*g*., 3–5]. Information from our study can inform future conservation policy and allow the Myanmar government to better assist local communities with HEC management, specifically in identifying indirect impacts that can be targeted for mitigation. In addition to mitigation strategies targeted towards reducing HEC in general, mitigation efforts that may alleviate the indirect impacts may consist of:

- increased access to medical, including psychological, services for families affected by HEC,
- efforts targeted at improving the quality of life for children in rural communities including access to school during times when elephants are present,
- subsidized construction materials and agricultural supplies to repair homes and replant crops.

Perhaps most importantly, our research shows that the entire community experiences indirect impacts, regardless of whether they have been impacted directly by HEC, and yet support for elephant conservation remains high. This indicates that the will to protect elephant species exists, if wildlife conservation and human-welfare agencies can find ways to provide the means for improved coexistence.

## Supporting information

S1 Fig (TIF)

S2 Fig (TIF)

S3 Fig (TIF)

S1 Appendix Summary of the number of participants (n = 381) that personally experienced each of the direct impacts used to calculate the direct impact score from surveys conducted in the Bago-Yangon and Ayeyarwady regions of Myanmar (May 2017-December 2018). (DOCX)
