# Supplementary figures and images for: A quantitative assessment of the indirect impacts of human-elephant conflict
Source: PLoS One. 2021 Jul 12;16(7):e0253784. doi: 10.1371/journal.pone.0253784 (PMC8274878; doi:10.1371/journal.pone.0253784)

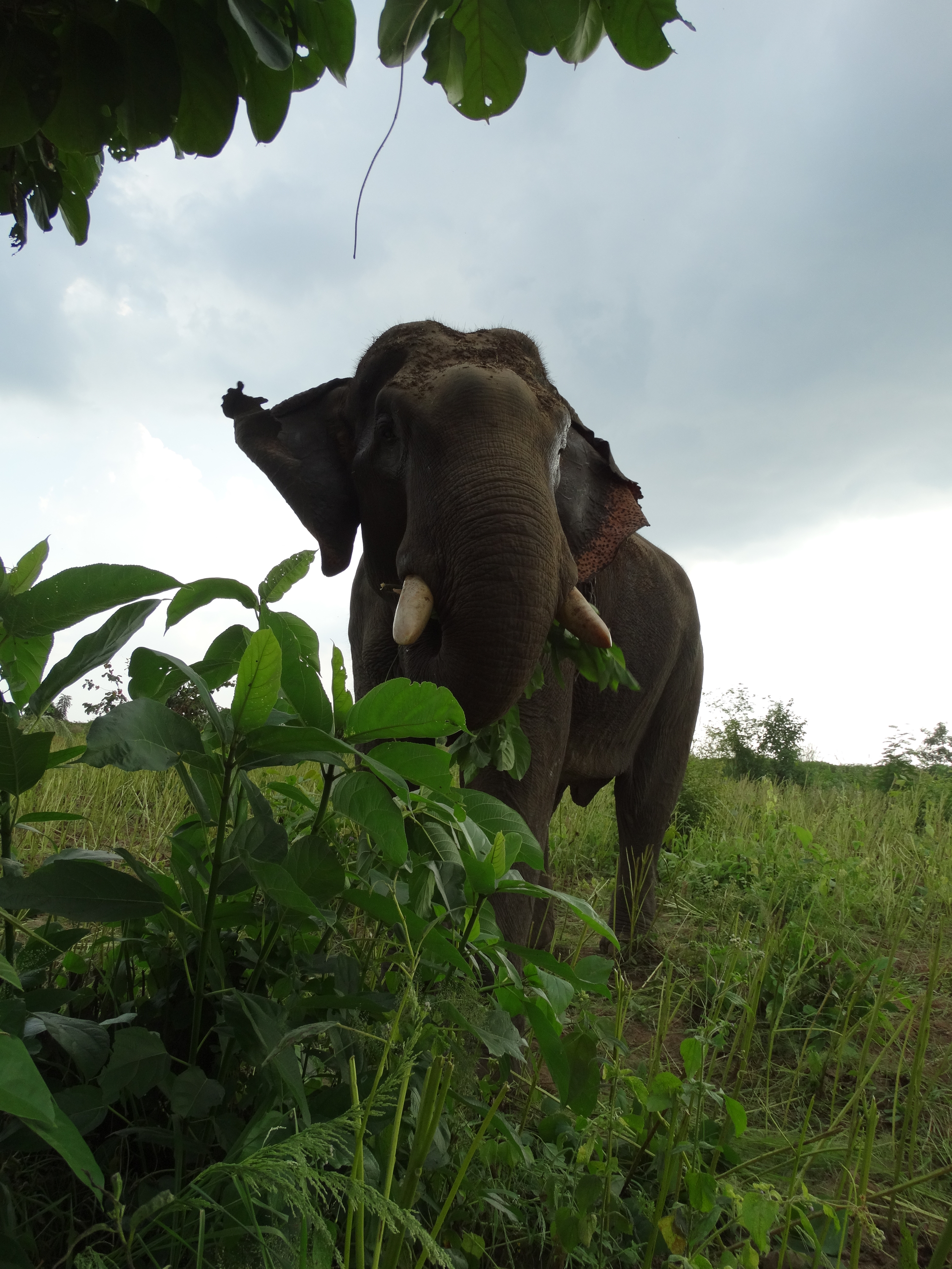

Supplement: S1 Fig — (TIF) [file pone.0253784.s001.tif]

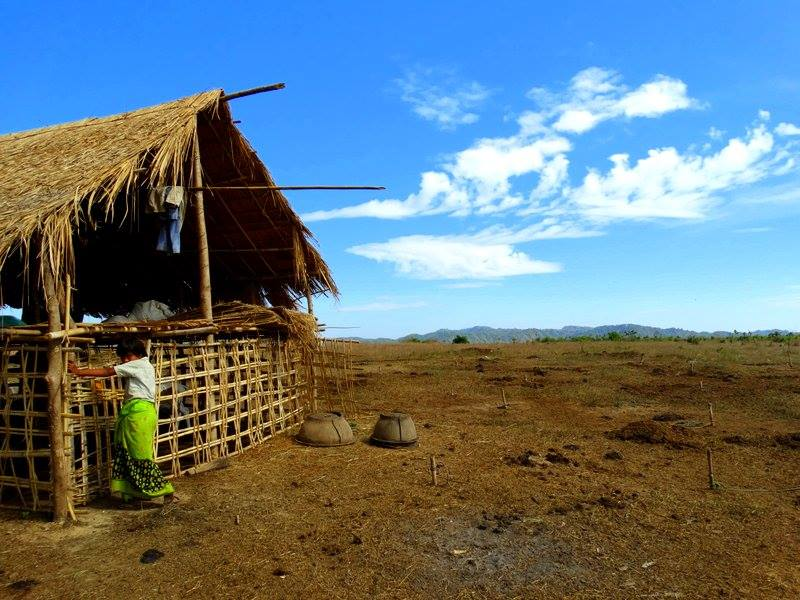

Supplement: S2 Fig — (TIF) [file pone.0253784.s002.tif]

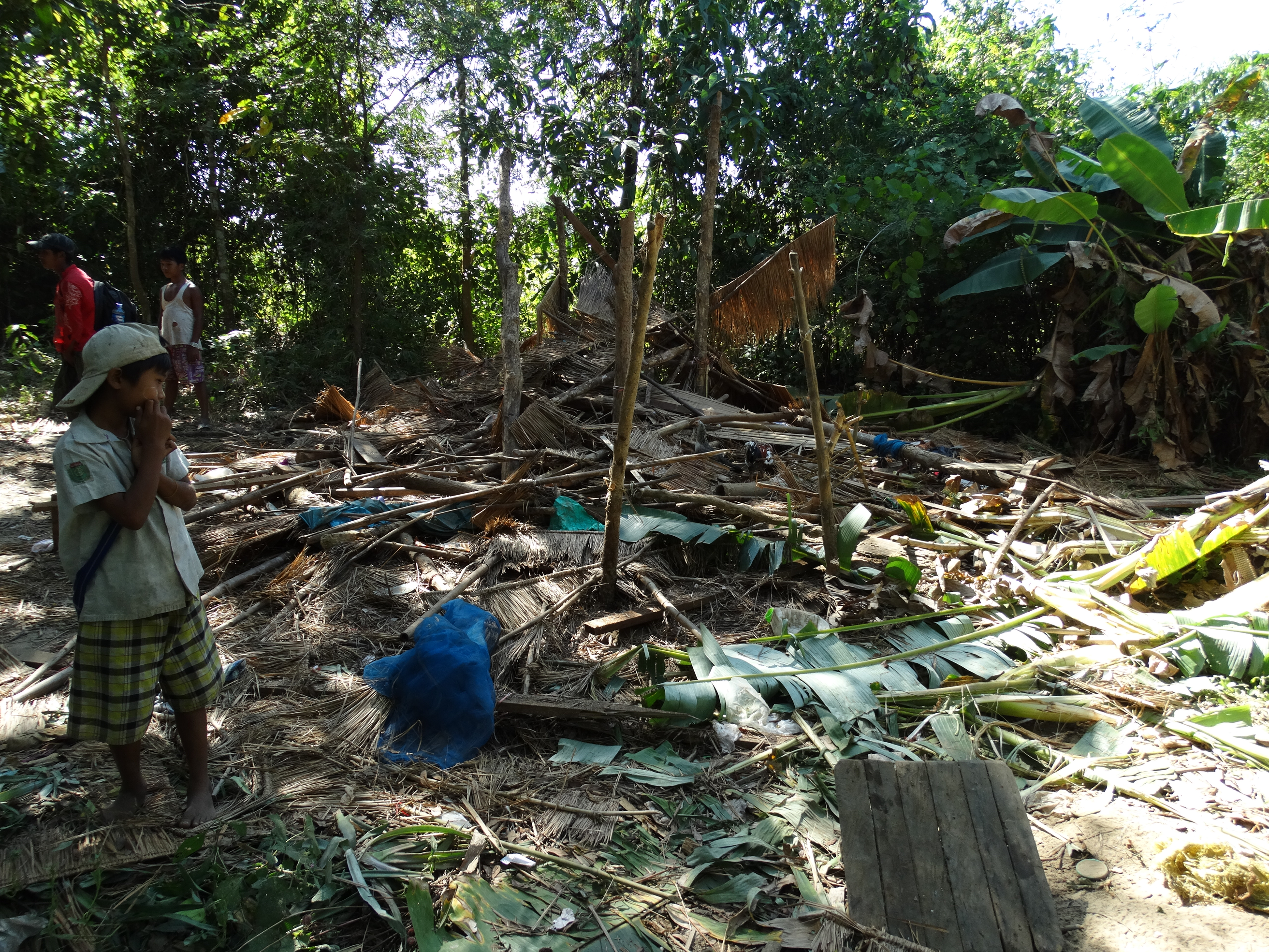

Supplement: S3 Fig — (TIF) [file pone.0253784.s003.tif]
