# Supplementary material for: A quantitative assessment of the indirect impacts of human-elephant conflict
Source: PLoS One. 2021 Jul 12;16(7):e0253784. doi: 10.1371/journal.pone.0253784 (PMC8274878; doi:10.1371/journal.pone.0253784)
Supplement: S1 Appendix — (DOCX) [file pone.0253784.s004.docx]

Appendix 1. Summary of the number of participants (n = 381) that personally experienced each of the direct impacts used to calculate the direct impact score from surveys conducted in the Bago-Yangon and Ayeyarwady regions of Myanmar (May 2017-December 2018).

| **Type of direct impact** | **Number of participants** | |
| --- | --- | --- |
| Been chased by an elephant | 129 | (34%) |
| Been injured by an elephant | 11 | (3%) |
| Had their home destroyed by an elephant | 53 | (14%) |
| Had their crops raided by an elephant | 198 | (52%) |
| Had livestock killed by an elephant | 5 | (1%) |
| Had other property destroyed by an elephant | 91 | (24%) |
| Had a family member injured or killed by an elephant | 19 | (5%) |
| Had someone they personally knew injured or killed by an elephant | 156 | (41%) |
